# Dictionary-enhanced imaging cytometry

**DOI:** 10.1038/srep43148

**Published:** 2017-02-22

**Authors:** Antony Orth, Diane Schaak, Ethan Schonbrun

**Affiliations:** 1 The Rowland Institute at Harvard, Cambridge, MA, 02141, USA; 2 ARC Centre of Excellence in Nanoscale BioPhotonics, RMIT University, Melbourne, VIC, 3001, Australia

## Abstract

State-of-the-art high-throughput microscopes are now capable of recording image data at a phenomenal rate, imaging entire microscope slides in minutes. In this paper we investigate how a large image set can be used to perform automated cell classification and denoising. To this end, we acquire an image library consisting of over one quarter-million white blood cell (WBC) nuclei together with CD15/CD16 protein expression for each cell. We show that the WBC nucleus images alone can be used to replicate CD expression-based gating, even in the presence of significant imaging noise. We also demonstrate that accurate estimates of white blood cell images can be recovered from extremely noisy images by comparing with a reference dictionary. This has implications for dose-limited imaging when samples belong to a highly restricted class such as a well-studied cell type. Furthermore, large image libraries may endow microscopes with capabilities beyond their hardware specifications in terms of sensitivity and resolution. We call for researchers to crowd source large image libraries of common cell lines to explore this possibility.

Optical microscopy is a core data gathering technique in the biological sciences. Digital microscopes enable researchers to record and store cellular scale images for further analysis offline^1^,^2^,^3^. Recent development of high throughput, gigapixel-scale microscopes^4^,^5^,^6^,^7^,^8^,^9^ have enabled collection of large datasets of cellular images. Once recorded, image processing routines turn this raw image data into quantifiable data, which can be used to aid diagnoses^10^ or to draw experimental conclusions^3^,^11^,^12^.

Often, the goal of a biological microscopy experiment isn’t to record the images themselves, but rather, to produce a low-dimensional quantification of the images^3^,^11^,^12^. While the input (the images) may contain a large amount of data, the output (abundance of each cell type, for example) takes the form of only a handful of scalar values. This mismatch suggests that there is a way to compress the input by using some *a priori* information about the cells. A compression of the input results in a faster image recording process, and decreased impact of phototoxicity and photobleaching. The type of *a priori* information used depends on the nature of the images. In cellular imaging experiments, one is often concerned with a restricted class of cells. These cells might differ slightly from one another in morphology, but they will share common features^13^,^14^,^15^,^16^,^17^. Armed with a reference library of typical cell images and associated properties, we may expect to be able to computationally reconstruct information about a cell from a severely undersampled or noise-corrupted input image^18^,^19^,^20^,^21^,^22^.

In this paper we explore how to leverage a large collection of reference images to enhance imaging cytometry. We use a high-throughput microlens array microscope^4^,^6^,^7^ to image and catalogue over 260,000 white blood cells (WBCs). WBCs are fluorescently labelled with a nuclear dye together with fluorescently tagged CD15 and CD16 markers. Cells are then mounted on a microscope slide for imaging. Individual cell images are extracted from raw fluorescence images using standard thresholding and segmentation approaches. Using CD15 and CD16 expression levels in each cell image, we show that the WBC population is divisible into 4 distinct populations, each with distinct nuclear morphologies. These populations are then gated to produce large dictionaries of nuclear morphology, which can in turn be explored. We demonstrate that these nuclear morphology dictionaries can be used to perform classification using only a fluorescence image of the nuclear shape. Moreover, this dictionary-based approach performs surprisingly well even in the presence of significant simulated imaging noise. This observation suggests that classification or quantification of cells in fluorescence microscopy can be done at low light levels given a library of example images that captures the full range of variability in cellular structure. Finally, we demonstrate dictionary-based denoising of nuclear morphology. We show that nuclear morphology can be accurately recovered from noisy images – even when the signal falls below the noise floor. This last result suggests a data-driven approach for ultra low light imaging. We discuss extensions of this idea, where crowd sourced high-resolution, multidimensionsal (3D, spectral, etc.) image dictionaries are used to enhance the capabilities of more rudimentary, cheaper microscopes.

## Results

### Imaging

**Figure 1:**
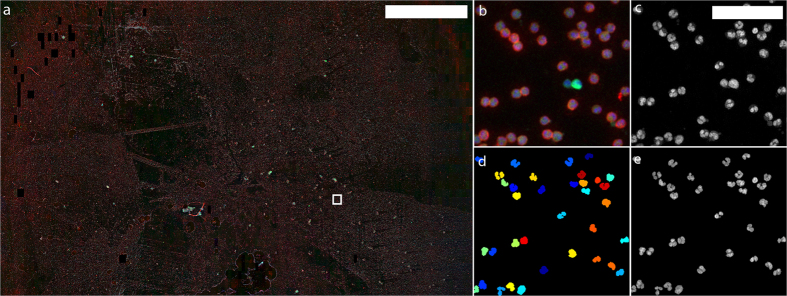
Large field-of-view imaging. (**a**) A cm-scale FOV of WBCs on a glass slide. WBCs are stained with a nuclear dye (Syto 16, blue), CD15 (APC, green) and CD16 (Brilliant Violet 421, red) markers. Dark patches are regions with high background fluorescence that have been discarded in post processing. Full-resolution version available online^35^. The entire image covers an area of roughly 1.7 × 1.1 cm. Scale bar is 3 mm. (**b**) Full-resolution image of the boxed region in (**a**). (**c**) Deconvolved image of the nuclear stain channel in (**b**). Scale bar is 50 μm. (**d**) Image in (**c**) after segmentation and size exclusion. Individual cells are outlined in colour for visualization. Each cell area is used as a mask for CD abundance integration. (**e**) Image (**c**) set to 0 in black region of the segmented image (**d**). These nuclear images are added to the image library.

We employ a high-throughput microlens microscope to image cm-scale areas of microscope slides, which are covered with fluorescently labelled WBCs as shown in Fig. 1a. This image covers an area of 1.7 × 1.1 cm at a sampling density of 0.5μm/pixel for a total size of ~0.75 gigapixels. We use 3 different fluorescent dyes to highlight the nuclei and quantify protein expression. Nuclei are dyed with Syto 16 (blue), while allophycocyanin (APC, green) and Brilliant Violet 421 (red) indicate CD15 and CD16 density, respectively. The microlens microscope operates like a highly parallelized point scanning microscope, producing an array of small sub-images – one for each microlens. When imaging continuous objects, these neighbouring sub-images are stitched together. However, because our goal is to build up a library of WBC images, we do not stitch sub-images from neighbouring microlenses. Instead, we apply a thresholding and segmentation routine to the Syto 16 channel for each sub-image independently in order to extract individual white blood cell nucleus images (Fig. 1b,c). Each WBC nucleus image identified by this process is entered into an array that makes up the library. The expression levels for CD15 and CD16 are calculated by summing the appropriate fluorescence channel within the WBC nucleus segmentation mask. An area normalized CD density can also be calculated, if desired. Segmenting only the nucleus as opposed the entire cell avoids ambiguity resulting from touching and overlapping cell membranes. White blood cell nuclei rarely overlap as they are well separated by the cytoplasm.

**Figure 2:**
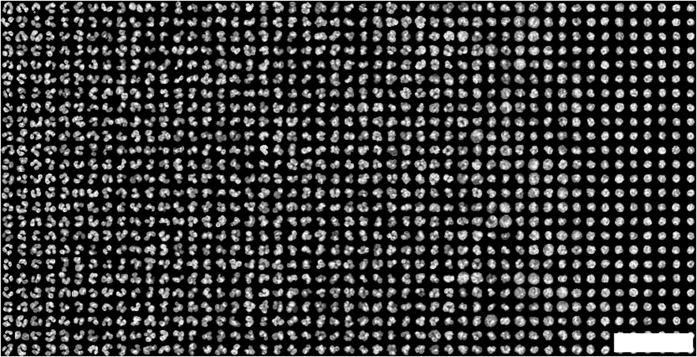
A portion of the white blood cell nuclear image library. Cells are organized from least to most circular (left to right). This image contains 1,225 cell nucleus images, slightly less that 0.5% of the complete dataset. Scale bar is 50 μm. The full version of this image containing 260,676 cells is available online^34^ and as Supplementary Data 1.

Using the WBC nucleus segmentation approach described in *Methods*, we extract 260,676 WBC nucleus images. Each nucleus image is 26 × 26 pixels in size (13 × 13 μm), with a CD15 and CD16 expression value for each cell. The entire set of extracted images is included as Supplementary Data 1. To aid in dataset exploration, we calculate a circularity parameter *C* for each WBC nucleus in the library 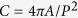. where *A* is the nuclear area and *P* is the nuclear perimeter. Figure 2 shows a snapshot of the roughly 0.5% of the entire WBC library (1,225 of 260,676 cells), organized least to most circular from left to right (low to high *C*). With this ordering one can quickly appreciate the diversity of WBC nuclear shapes present in the library. Indeed, other orderings, such as by eccentricity, total area or CD expression are also possible.

### Classification

In this section we show that nuclear morphology can be used directly to classify WBCs. We first identify 4 distinct gates emergent from our CD15/CD16 labelling approach. These gates are then split into dictionary and trial sets for correlation-based classification.

**Figure 3:**
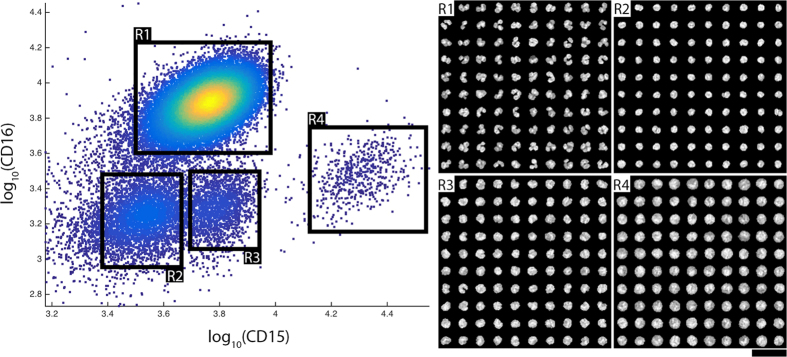
CD15/16 expression and cellular morphology. Log-scale CD15 (x-axis)/CD16 (y-axis) scatter plot of WBCs. WBCs are gated into four regions, denoted R1-R4. Insets: 100 representative nuclear cell images for each region. Differing morphology in each gate is readily apparent. Scale bar: 25 μm.

Four distinct clusters are visible upon displaying a subset (see *Gating* in *Methods*) of the cellular CD15/CD16 expression data as a log-scale dotplot (Fig. 3). We gate each one of these clusters manually into gates labelled R1-R4. A sampling of the nuclear images in each gate is shown in the insets to Fig. 3. We will refer to the entire set of nuclear images in gates R1-R4 as the reference library. The reference library is composed of 23,894 R1; 3,583 R2; 1,685 R3; and 517 R4 images.

One can clearly see the distinctive multi-lobed nuclei characteristic of neutrophils in R1^23^. The population in gate R2 is likely dominated by lymphocytes, as evidenced by the small circular nuclei found in R2. Nuclei in R3, most likely belonging to a CD16- monocyte population, are slightly bigger and less circular than those in R2^24^. Finally, there is a small number of large, highly expressing CD15++ cells in gate R4. These cells are possibly undifferentiated granulocytes, however, further investigation is necessary for unambiguous identification. Other types of less common WBCs such as basophils, eosinophils and natural killer cells will be found throughout these gates^24^, but are unresolvable with our two-dimensional CD15/CD16 labelling strategy.

Next, we implement an image-matching WBC classification scheme. The approach is as follows. From each gate, we remove 100 cells from the reference library to construct a trial image set. The remaining reference images we refer to as the dictionary. We then proceed to classify each cell in the trial set as belonging to one of R1-R4 based solely on the magnitude of the cross-correlation with nuclear images from the dictionary. By knowing the ground truth gate of each cell in the trial set, we can quantify the accuracy of the classification scheme. Each of the 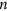 images in the trial set is cross-correlated with each of the 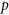 images in the reference set (across all gates), yielding a matrix 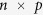 image correlation values. Cross-correlation values range from 0 to 1, with 1 indicating a perfect match, and 0 signifying no similarity. For each cell in the trial set, a portion of the top correlation values for each cell type (R1-R4) is averaged, yielding a *similarity index* for each cell type. The optimal number of top correlation values to include in this average depends on the dictionary size and morphology variation (see Supplementary Note 4 and Supplementary Figures 1–3). The trial cell is classified as being of the cell type with the highest similarity index value.

**Figure 4:**
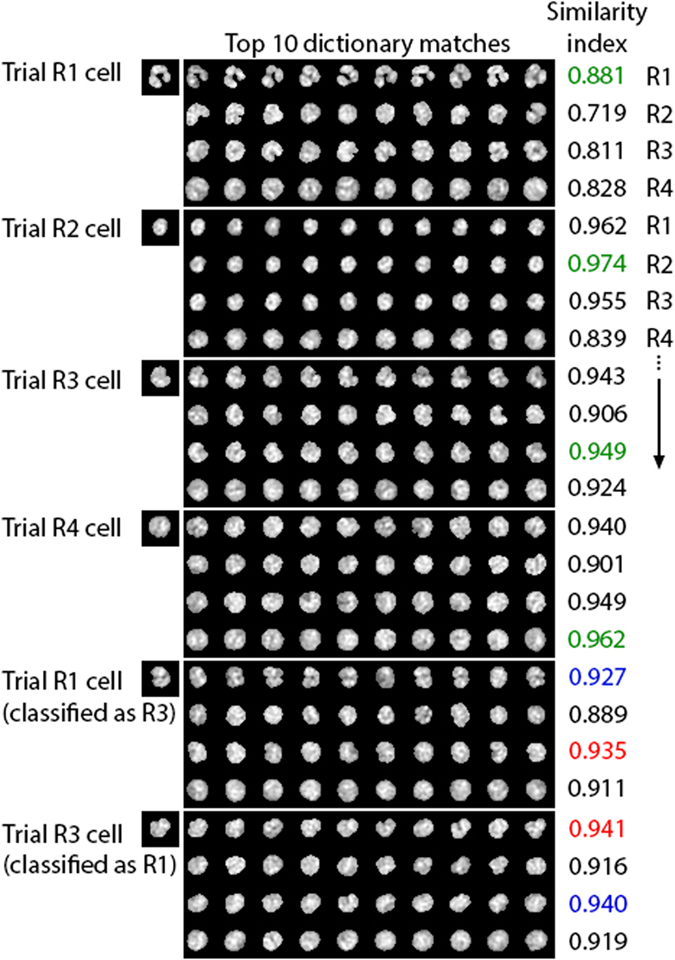
Typical examples of top 10 dictionary matches for each cell type. The first 4 trial cells (R1-R4) are correctly classified as R1-R4 cells. The bottom two trial cells are examples of incorrectly classified trial cells (R1 classified as R3 and R3 classified as R1). The similarity indices for each cell type (R1-R4) is shown in the “Similarity index” column. Green text indicates a correct match, red indicates an incorrect match and blue indicates the correlation value of the correct cell type.

Examples of trial cells and their 10 most highly correlated reference cells of each type are shown in Fig. 4. Note that while there are sometimes closely matching images for incorrect cell types, usually there are too few reference cells of the wrong type with high correlation values to result in a false classification. This feature makes our method robust against poorly classified reference data. A pair of examples of incorrect matches between R1 and R3 gates is also shown in Fig. 4. We comment on the nature of the crosstalk between these gates below. The entire distribution of correlation values for the correctly classified trial cells in Fig. 4 are shown in Supplementary Figure 4. Another way of looking at the correlation data is shown in Supplementary Figures 5–7, where we plot the decrease in correlation value in order of the correlation ranking.

To evaluate the accuracy of our classification algorithm, we applied the algorithm to 50 random permutations of trial sets and dictionaries. To calculate the cell similarity indices, we averaged the correlation values for the top *m* = 17 matches for R1, R2 and R4 dictionaries, while the R3 cell similarity index was obtained by averaging over the top *m* = 9 matches in the R3 dictionary. The number of top matches used (*m*), to calculate the cell similarity index for each dictionary (*m* = 17 for R1, R2, R4 and *m* = 9 for R3), was arrived at by calculating the overall classification accuracy for different numbers of top matches as outlined in Supplementary Note 4 and Supplementary Figure 1. Classification results are displayed as a confusion matrix in Table 1. Cells from gates R1, R2 and R4 are classified with over 90% accuracy. Cells from gate R3, however are classified correctly at a lower rate of 85.26%. Overall, 90.15% of trial cells are correctly classified. For comparison, the classification accuracy using a quadratic discriminant analysis (QDA)^25^, with a 5-dimensional feature vector (cell nucleus area, perimeter, circularity, eccentricity and solidity) is shown in Supplementary Table 4. The overall classification accuracy of this more traditional approach is 85.31%, nearly 5% less than with the dictionary-based approach. The dictionary-based method performs similarly to QDA for R2-R4 cells, but greatly outperforms QDA for R1 cell classification: 90.06% vs. 63.04% for dictionary-based and QDA, respectively.

We note that the lower classification accuracy of R3 is likely a result of monocytes being present in both R3 and R1 gates. It is well known that there is a ~10–20% non-classical CD16+ monocyte population in in the peripheral blood of a healthy donor^26^. Assuming these CD16+ monocytes have similar nuclear morphology to CD16- monocytes, they will result in cross talk between the R1 and R3 gates. This would happen both due to CD16+ monocytes in R1 matching with CD16- monocytes is R3, and vice-versa.

**Figure 5:**
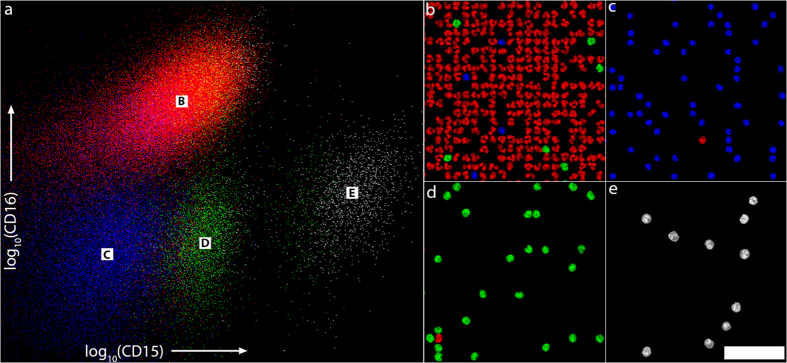
Cell classification visualization. (**a**) Dotplot showing locations of a portion of the dataset (82,000 cells). Cells are color coded according to nucleus-based classification: Cells classified as R1, R2, R3 and R4 are displayed in red, blue, green and white, respectively. (**b–e**) Zoom in of regions marked B-E in (**a**). Scale bar is 50 μm. A full-resolution version of (**a**) is available online^27^. Of the 82k cells shown here, 66.91% are classified as R1, 25.07% are classified as R2, 6.09% are classified as R3, and 1.93% are classified as R4.

We applied the classification algorithm to a subset of 82,000 cells from our dataset. This subset is roughly 38% of the entire dataset and does not include any dictionary cells. The results are visualized in a color-coded dotplot in CD15/CD16 expression space, where at each datapoint we display a typical WBC nucleus image with the appropriate CD15 and CD16 expression (Fig. 5a). A larger version of this image available online allows the user to zoom in to discover the nuclear morphology in CD15/CD16 expression space^27^. Typical regions within R1-R4 gates are shown at a larger magnification in Fig. 5b–e. Of this 82,000 trial cell set, 66.91% are classified as R1, 25.07% as R2, 6.09% as R3 and 1.93% as R4. The proportions of R1, R2 and R3 classified trial cells are roughly in line for the expected abundance of neutrophils, lymphocytes and monocytes in peripheral blood^28^,^29^. Roughly 15% of the cells classified as R3 are located within the R1 gate. This matches well to the known normal range for the percentage of CD16+ monocytes in peripheral blood^26^, lending weight to our hypothesis that the R1/R3 crosstalk is due to a CD16+ monocyte population.

Typical approaches to cell classification rely on segmentation followed by extracting morphological features such as area, perimeter and curvature. However, these procedures become increasingly difficult to perform in noisy conditions. In contrast, our method avoids the need for precise segmentation and feature extraction by performing the classification process by direct image correlation with a low-noise reference set. We simulate this process by adding Gaussian noise to trial image sets in post-processing prior to cell classification. Classification results for a trial image set with a signal-to-noise-ratio (SNR) =3.5 are shown in Table 2. Even in the presence of significant noise, R2 and R4 cells are still classified with over 91% accuracy, while R1 and R3 cells are classified with 85.90% and 77.36% accuracy, respectively. At this low SNR, there is only a minor drop in the overall classification accuracy from the SNR = 90 case: 90.15% to 86.97%. The optimal number of top matches to include in calculation for the cell similarity indices was significantly larger than in the SNR = 90 case. For optimal classification at SNR = 3.5, we averaged over the top (69, 81, 21, 9) correlation values for (R1, R2, R3, R4) dictionaries to calculate the cell similarity indices (see Supplementary Figure 2).

The classification results in Table 2 are compared to QDA classification on the same noisy data in Supplemental Table 2. Our image matching approach matches or outperforms QDA for all cells types. The largest difference is for R1 cells, where the classification accuracy using segmentation and QDA on noisy images drops to 57.28% compared with 85.90% with image correlation. This large performance gap is a result of unreliable segmentation under noisy imaging conditions. The overall classification accuracy for SNR = 3.5 data is 86.97% with the dictionary-based method, compared to 75.67% using QDA. The performance gain of the dictionary-based method is even more pronounced for extremely low SNR values. Supplemental Tables 3 and 4 show that the dictionary-based method still retains an accuracy of 78.22% at a SNR = 1.75 in comparison to 57.46% using QDA. Comparisons for lower SNR values were not feasible due to unreliable image segmentation required to calculate the morphological features for QDA.

### Denoising

In addition to high classification accuracy at low SNR values, the reference library also provides a means of denoising. By simply taking the average over the most highly correlated cells in the entire nuclear image dictionary, we can construct denoised estimate of the original image. To increase the size of the library, thereby increasing the likelihood of good dictionary matches, we also include copies of each cell rotated in 30**°** intervals over 360**°**. In this case there are over 3 × 10^6^ cell nucleus images in the dictionary.

**Figure 6:**
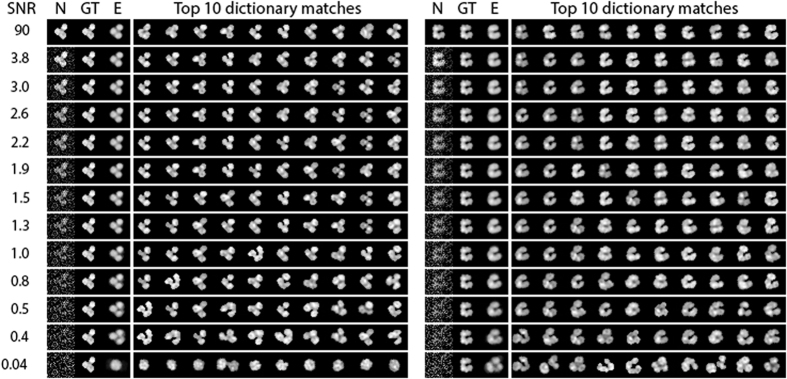
Dictionary-based denoising of R1 nucleus images. The left column (N) shows a noisy nucleus image with SNR levels as indicated in each row. The original ground truth (GT) trial image (SNR = 90) is shown in the “GT” column and the denoised estimate (“E”) column shows the average of the top 10 dictionary matches for that trial cell. Even at low SNR, basic morphology of the nucleus can be recovered.

In Fig. 6, we show two examples of denoising for R1 cells where we simulate noisy imaging in post-processing. Cell nucleus images are corrupted with Gaussian noise of fixed variance, while the signal (ie. the pixel values of the original images) is scaled down, resulting in images with low SNR. Noise corrupted images are shown under columns labelled “N” in Fig. 6, and the ground truth images (pre-noise corruption) are found in the “GT” column. The top 10 dictionary matches are also indicated. The estimated cell image, resulting from averaging over the top 10 dictionary matches is shown in the “E” column. There is little change in the estimated cell image from SNR = 90 (the original cell nucleus image with no extra noise) down to 0.5. Remarkably, the de-noised estimates still reveal the general structure of the cell nucleus in cases where the signal is buried below the noise level. Estimates become increasingly unreliable below this level. This result is made possible because of a highly restricted set of WBC nuclear images and the large dictionary set. Image details below the noise floor consistently lead to appropriate matches given a suitable dictionary.

## Discussion

We have demonstrated an approach to data-enhanced WBC cell classification and image denoising. Our direct image correlation approach maintains high cell classification accuracy even in the presence of high noise. Moreover, we are able to obtain morphologically accurate denoised estimates of WBC nucleus images below the noise floor at an SNR = 0.5, which is 180-fold lower than the SNR of the original dataset. When fixed detector noise is the dominant form of signal corruption, recovering an acceptable image from a 180-fold lower SNR in principle allows for a 180x higher frame rate or 180x less excitation dose (assuming read-noise dominated imaging). The latter is of utmost importance when imaging live cells or sparsely labelled samples. Decreased irradiation is also critical in x-ray computed tomography (CT) imaging. Our results suggest that a similar dictionary based approach may be valuable in other imaging fields and need not be confined to fluorescence microscopy of WBCs. We note that in our denoising and noisy classification simulations, noise was added after initial cell localization. A real low-light experiment will not have this luxury and will require precise identification of cell positions within noisy microscope images. This is a similar problem to localization of single molecule fluorescence in super resolution techniques^30^, which can be achieved in the presence of significant noise.

Though we have applied our technique to WBCs, we expect that the direct image correlation method we present here will be applicable to a wide variety of cell types when paired with large image libraries. Experiments involving standard cell lines that are used widely in the scientific community may benefit significantly from this approach. For example, an image library of HeLa cells^15^,^31^,^32^ might enable extremely low excitation intensity fluorescence imaging of this model cell line, thereby sidestepping limitations arising from photobleaching and phototoxicity.

Given the widespread use of certain cell lines, it should be possible to build up massive image databases by crowd sourcing the library collection task. Such a freely available database would have implications beyond what we have demonstrated with classification and denoising applications. Using our data-driven approach, researchers with low-resolution imaging hardware might be able to use a high-resolution image database to estimate a super-resolved version of their data. Researchers may be able to estimate cellular structure across dimensions as well. If a large 3D reference set (eg. confocal image stack) of cell type were available, a 2D slice of a trial cell could be sufficient to locate a match in the reference set, thus obtaining an estimate for 3D structure of the cell. Ultimately, such imaging estimates are only useful if they are able to predict a certain property of a cell, such as its protein expression or metabolic state. We have demonstrated this by showing that image-based classification can reproduce manual gating of CD15/CD16-labeled WBCs.

Constructing appropriately large reference libraries is a time consuming task best suited to high throughput microscopes, such as the one employed in this work or imaging flow cytometers^33^. As these speciality hardware systems become more widely available, users will have the opportunity to assemble open source image libraries to be shared amongst the scientific community. Those without access to high throughput systems could then use these libraries as references for *a priori* data-enhanced operations such as cell classification, denoising. In this vein, we are releasing the reference libraries acquired for this work, as Supplementary Data 1. The image sets are also available in a web friendly format on Gigapan^27^,^34^,^35^. We encourage researchers to explore the images in this dataset, and in particular to observe the fascinating array of nuclear shapes in the complete dataset.

## Methods

### Slide preparation

Microscope coverslips (#1) used for sample imaging were prepared by applying a thin layer of 0.5% porcine gelatin, air dried, and then rinsed with water. A thin layer of 1% BSA in PBS was then applied to the porcine layered coverslips, air dried, and rinsed with water.

### White Blood Cell Isolation and Staining

10 ml EDTA preserved fresh blood drawn 1–2 hours prior to cell preparation was purchased from Research Blood Components, LLC (Brighton, Ma). Red blood cells were removed for the sample using a RBC lysis buffer protocol provided by the company (Alfa Aesar), leaving the white blood cell population whole and intact. The final white blood cell concentration was adjusted to 1 × 10^6^ in 100 μl for each slide prepared. This 100 μl sample volume included 20 μl APC-Mouse Anti-Human CD15 antibody (BD-BioSciences), 5 ul Brillant Violet 421-Mouse Anti-Human CD16-antibody (BD Biosciences), and 2 μl 0.1 mM Syto 16 Green Fluorescent Nucleic Acid Stain (ThermoFisher). DMEM (Lonza BioWhittaker 4.5 g/L glucose without Glutamine or Phenol Red) was added to samples to bring volume to 100 μl for each white blood cell sample. White blood cell samples with antibodies were mixed on a rotator for 30 minutes at room temperature, then rinsed in DMEM buffer, and spun down at lowest speed possible to remove unbound antibody. 1% PFA was added and incubated at room temperature for 20 minutes to fix cells. Labelled cells were rinsed with DMEM and spun out of solution at lowest speed possible to separate out the PFA. Labelled cells were resuspended in 50 μl DMEM buffer and spread evenly onto prepared coverslips, and then air-dried. Mowiol mounting solution (Calbiochem) was layered over cells on coverslips, and coverslip was mounted onto a microscope slide. These slides were placed in a dark place overnight at room temperature to allow the Mowiol to harden. Slides with prepared labelled cells were stored in the dark until imaged.

### Imaging

Samples were imaged using a high throughput microlens array microscope^4^,^6^. Plano-convex refractive microlenses are fabricated by replica molding a photoresist master into Norland Optical Adhesive 61 (n = 1.56). Microlenses are arranged in a hexagonal grid, with focal length 300 μm and diameter 122 μm. Brilliant Violet, Syto 16 and APC channels were imaged sequentially by exciting with 405, 488 and 647 nm lasers, respectively. The total excitation power at the microlens array is roughly 65 mW for each laser. Long pass emission filters were employed together with a quad-band dichroic beam splitter to eliminate laser scatter. The excitation laser beam is expanded and projected into a weakly diverging 2 × 2 cm square on the flat side of the microlenses. Each microlens focuses the incident light into a spot at its focus. The sample is placed at the focal plane of the microlens array, resulting in fluorescence emission. This fluorescence is captured by each microlens, and directed back towards a large single lens reflex (SLR) lens (Nikon, focal length = 50 mm, f/1.4). The SLR lens images the back aperture of all microlenses onto a fast CMOS camera running at 200 frames per second (Point Grey Grasshopper 3 GS3-U3-23S6M-C). To build up a point scanning image for the portion of the sample under each microlens, the sample is raster scanned over a 130 × 118 μm under the microlens array, at a sampling frequency of 0.5 μm/pixel. The fluorescence emission at any given point in the image is given by the brightness of the pixels that make up the image of the microlens on the camera sensor.

### Data Processing

After imaging, all subsequent data and image processing is performed in MATLAB. Transforming the raw point scanning data into 2D images first requires the position of each microlens in the array to be identified. A reference microlens array image is constructed by taking the average of the raw camera sensor image over 1000 frames. The periodicity of the hexagonal microlens array is obtained by computing the 2D Fourier transform of the reference image, and locating the peak positions in the spatial frequency domain. A digitally constructed template hexagonal grid with identical periodicity to the reference image is then cross-correlated with the reference image. The peak cross correlation value within one hexagonal cell indicates the necessary translation of the template to match the reference image. The location of the centre of each hexagonal cell in the template grid is indexed with a microlens identification number, so that pixels in the reference image can be linked directly to a microlens index. The sub-image recorded by each microlens is extracted by arranging pixel intensities in lines, with 260 pixels (130 μm) per line.

### Image Processing

Sub-images with large background (large minimum intensity values) are discarded. The image recorded at each microlens position is thresholded using Otsu’s method to isolate WBC nuclei. The resulting binary image is further subjected to a variety of MATLAB morphological operations to clean object boundaries (MATLAB functions *bwareaopen* and *bwmorph*, operations used: ‘bridge’, ‘diag’, ‘spur’, ‘hbreak’, ‘clean’, ‘remove’, ‘thicken’, ‘holes’). The resulting binary image is shown in Fig. 1d, with each object (WBC nucleus) labelled by colour. Each isolated region of Fig. 1d is used as a mask for a WBC nucleus image. Eccentricity, perimeter, area and solidity of each WBC nucleus region are calculated using the MATLAB *regionprops* function. CD15 and CD16 expression are calculated by summing the fluorescence intensity in the appropriate spectral channel over the region of each WBC nucleus mask. CD expression data can vary due to intrinsic brightness variation of the microlens image on the image sensor. This can be due to inaccurate microlens position identification or microlens efficiency variation throughout the array. This effect is corrected at two points in the image-processing pipeline. First, the intensity of each microlens FOV is scaled so that all FOVs have identical mean values (only pixels within cells are considered when calculating the mean). This correction is performed prior to segmentation. The second correction occurs after cell image and CD expression extraction. Because cell image extraction proceeds in a row-wise fashion across the entire FOV, any systematic spatial bias of CD expression levels is apparent when plotting CD expression against cell number (the first cell extracted is cell number 1, the next cell is cell number 2 and so on). This spatial bias is due largely to the angle between the pixel rows on the camera and the rows of microlenses. This effect manifests itself as periodic variation in CD expression as the cells are extracted from microlens FOVs that snake from top left to bottom right of the cm-scale large FOV. To correct for this bias, we low pass filter the CD expression trace (ie. the plot of CD expression vs. cell number) such that intra-FOV CD expression variation (which is what we want to capture) is averaged out, leaving only the systematic inter-FOV variation. Then, the original CD expression trace is divided by the low-pass filtered trace, eliminating systematic intensity variations. When normalized to its highest value, we refer to the low pass filtered trace as the intensity correction factor. This factor is used in the gating process to establish the quality of the dictionary images. This process is performed for both CD15 and CD16 channels.

### Gating

WBC images containing nuclei from two different WBCs are identified and eliminated by gating out cellular populations with large area and large eccentricity. For dictionary construction, we further narrow down the WBCs used. We discard all WBCs found in fields-of-view where the intensity correction factor is greater than 1.33. The dotplot in Fig. 3 shows only datapoints corresponding to cells with an intensity correction factor less than 1.33. This ensures that the WBCs in the dictionary have highly accurate CD expressions values (larger correction values come with higher uncertainty), and therefore that they are found in the correct gate. Remaining WBCs are subsequently gated into R1-R4 populations based on CD15/CD16 expression

### Noise Simulation

For noisy classification and denoising examples, noise is added to the original cell nucleus images (ground truth) using the MATLAB “imnoise” function. The SNR of the original dataset is 90, calculated by taking the mean pixel values within a cell, divided by the standard deviation of the pixel values in background regions. The SNR is calculated in the same way for synthetic noisy images.

### Quadratic Discriminant Analysis Classification

As a comparison to a traditional classification technique, we use Quadratic Discriminant Analysis^25^ to classify cell nuclei based on their area, perimeter, circularity, eccentricity and solidity. These 5 properties form a 5-dimensional feature vector for each cell based on segmentation from the background. Segmentation proceeds as in “Image Processing”.

## Additional Information

**How to cite this article:** Orth, A. *et al*. Dictionary-enhanced imaging cytometry. *Sci. Rep.* **7**, 43148; doi: 10.1038/srep43148 (2017).

**Publisher's note:** Springer Nature remains neutral with regard to jurisdictional claims in published maps and institutional affiliations.

## Supplementary Material

Supplementary Information

Supplementary Dataset 1

## Figures and Tables

**Table 1:** Confusion matrix for cell classification resulting from 50 randomized trial and dictionary dataset pairs.

	Identified as R1	Identified as R2	Identified as R3	Identified as R4
R1	4503 (90.06%)	91 (1.82%)	383 (7.66%)	23 (0.46%)
R2	255 (5.10%)	4590 (91.80%)	155 (3.10%)	0 (0.00%)
R3	346 (6.92%)	248 (4.96%)	4263 (85.26%)	143 (2.86%)
R4	0 (0.00%)	0 (0.00%)	327 (6.54%)	4673 (93.46%)

Cells within each trial dataset are classified based on their correlation to the dictionary datasets. Trial datasets consist of 100 cells in each of R1-R4 gates. The dictionary dataset is composed of 23,794 R1; 3,483 R2; 1,585 R3 and 417 R4 cells. Classification is based on the average of the top *m = (17, 17, 9, 17)* dictionary matches for (R1, R2, R3, R4) dictionaries. The overall classification accuracy is 90.15%.

**Table 2:** Classification in the presence of noise.

	Identified as R1	Identified as R2	Identified as R3	Identified as R4
R1	4295 (85.90%)	130 (2.60%)	454 (9.08%)	121 (2.42%)
R2	251 (5.02%)	4591 (91.82%)	153 (3.06%)	5 (0.10%)
R3	472 (9.44%)	285 (5.70%)	3868 (77.36%)	375 (7.50%)
R4	4 (0.08%)	0 (0.00%)	356 (7.12%)	4640 (92.80%)

Trial cell images are corrupted with Gaussian noise down to a signal-to-noise-ratio (SNR) of 3.5. The dictionary dataset is composed of 23,794 R1; 3,483 R2; 1,585 R3 and 417 R4 cells. Classification is based on the average of the top *m = (69,81,21,9)* dictionary matches for (R1, R2, R3, R4) dictionaries. The overall classification accuracy is 86.97%.
