# Supplementary material for: Dictionary-enhanced imaging cytometry
Source: Sci Rep. 2017 Feb 22;7:43148. doi: 10.1038/srep43148 (PMC5320489; doi:10.1038/srep43148)
Supplement: Supplementary Information [file srep43148-s1.pdf]

Dictionary-enhanced imaging cytometry

Antony Orth[1,2,*], Diane Schaak[1] and Ethan Schonbrun[1]

[1]The Rowland Institute at Harvard, Cambridge, MA, USA 02141
[2]ARC Centre of Excellence in Nanoscale BioPhotonics, RMIT University, Melbourne, VIC, Australia 3001
*antony.orth@rmit.edu.au

Supplementary Information

Supplementary Note 1. The entire set of 260,676 white blood cell nucleus images is included in MATLAB format as Supplementary Data 1. The data are contained in a 26x26x260676 *uint8* variable called "cells" in SUPP_DATA_1.mat. To view cells as a movie, open the SUPP_DATA_1.mat and execute the command *implay(cells)*.

Supplementary Note 2

To aid comparison between our image-based correlation method and classical classification schemes, we performed classification of WBCs based on quadratic discriminant analysis (QDA). Briefly, this technique partitions the feature space with second-order polynomial boundaries. This is more general version of linear discriminant analysis, which partitions the n-dimensional feature space using n-1 dimensional planes. In our case, we use a 5-dimensional feature space consisting of WBC nucleus area, circularity, and perimeter, solidity and eccentricity. The results of the classification, for both low noise (SNR=90, Supplementary Table 1) and high noise (SNR=3.5, Supplementary Table 2) scenarios are presented below. Supplemental Table 1 should be compared to Table 1 in the main text and Supplemental Table 2 should be compared to Table 2 in the main text. In both cases, our dictionary-based method outperforms QDA.

	Identified as R1	Identified as R2	Identified as R3	Identified as R4
R1	3197 (63.94%)	323 (6.46%)	1460 (29.20%)	20 (0.40%)
R2	138 (2.76%)	4672 (93.44%)	190 (3.80%)	0 (0.00%)
R3	220 (4.40%)	257 (5.14%)	4368 (87.36%)	155 (3.10%)
R4	0 (0.00%)	0 (0.00%)	176 (3.52%)	4824 (96.48%)

Supplementary Table 1. Confusion matrix for quadratic discriminant analysis with SNR=90. The overall classification accuracy is 85.31%.

	Identified as R1	Identified as R2	Identified as R3	Identified as R4
R1	2864 (57.28%)	443 (8.86%)	1397 (27.94%)	296 (5.92%)
R2	248 (4.96%)	4508 (90.16%)	239(4.78%)	5 (0.10%)
R3	450 (9.00%)	399 (7.98%)	3770 (75.40%)	381 (7.62%)
R4	219 (4.38%)	2 (0.04%)	777 (15.54%)	4002 (80.04%)

Supplementary Table 2. Confusion matrix for quadratic discriminant analysis classification with SNR=3.5. The overall classification accuracy is 75.72%.

Supplementary Note 3

Confusion matrices for classification using both our dictionary-based method and QDA for an SNR of 1.75 are shown in Supplementary Tables 3 and 4, respectively. At such a low SNR, the overall classification accuracy of our dictionary-based method outperforms QDA by over 20%.

	Identified as R1	Identified as R2	Identified as R3	Identified as R4
R1	4067 (81.34%)	268 (5.36%)	529 (10.58%)	136 (2.72%)
R2	313 (6.26%)	4482 (89.64%)	204 (4.08%)	` (0.02%)
R3	1085 (21.70%)	353 (7.06%)	3256 (65.12%)	306 (6.12%)
R4	168 (3.36%)	7 (0.14%)	987 (19.74%)	3838 (76.76%)

Supplementary Table 3. Confusion matrix for dictionary-based correlation classification with SNR=1.75. The overall classification accuracy is 78.22%. Classification is based on the average of the *m=(97,53,13,5)* top dictionary matches for (R1, R2, R3, R4) dictionaries. The overall classification accuracy is 78.22%.

	Identified as R1	Identified as R2	Identified as R3	Identified as R4
R1	1911 (38.22%)	999 (19.98%)	1402 (28.04%)	688 (13.76%)
R2	817 (16.34%)	3439 (68.78%)	695 (13.90%)	49 (0.98%)
R3	522 (10.44%)	765 (15.30%)	2554 (51.08%)	1159 (23.18%)
R4	302 (6.04%)	109 (2.18%)	1001 (20.02%)	3588 (71.76%)

Supplementary Table 4. Confusion matrix for quadratic discriminant analysis classification with SNR=1.75. The overall classification accuracy is 57.46%.

Supplementary Note 4

Optimal classification accuracy using our dictionary-based method requires a proper choice of operating parameters. Specifically, when classifying a trial cell, we average the correlation value of the trial cell with the top *m* matches in each dictionary set to calculate the cell similarity index. How to choose how many *m* top matches to include in the average for the cell similarity index? In general, the optimal *m* value will be different for each cell type dictionary. The number of top matches to use will depend on the intrinsic morphology variability of the cell type and on the number of cells images in the dictionary. To identify the optimal number of matches to consider for each dictionary type, we run 3 replicates of the classification scheme and calculate the percentage of correctly classified cells for different *m* values. Due to the large dimensionality of this parameter space, we consider only every 4th *m* value up to 97 (ie. m =1, 5, 9, ..., 97). The entire parameter space is 4-dimensional with 25x25x25x25 entries.

Below, we show planes from the 4-D parameter space showing maximum classification accuracy for 3 particular SNR values (SNR = 90, 3.5 and 1.75 for Supplementary Figures 1-3, respectively). Each 2-D plane shows the variation in classification accuracy as a function of the number of top R1 matches used and of the number of top R3 matches used to calculate the cell similarity index. In general, classification is better when using a smaller number of top matches to calculate the cell similarity index for dictionaries with fewer entries. For example, the R3 dictionary has only 1,585 entries, whereas the R1 dictionary has 23,794 entries. Consequently, the optimal number of top R3 matches to use when calculating the cell similarity index is always smaller than the number of top R1 matches used.

Note that the classification accuracies reported in Supplementary Figures 1-3 are obtained over 3 randomized trials of the classification scheme. The optimal classification error value from these supplementary figures may differ from that reported in the text because those in the text are calculated from 50 randomized trials of the classification scheme. We used 3 randomized trials to calculate the classification error over the 4-dimensional parameter space to minimize calculation time. However, using 50 randomized trials results in a more accurate estimate of the classification accuracy.

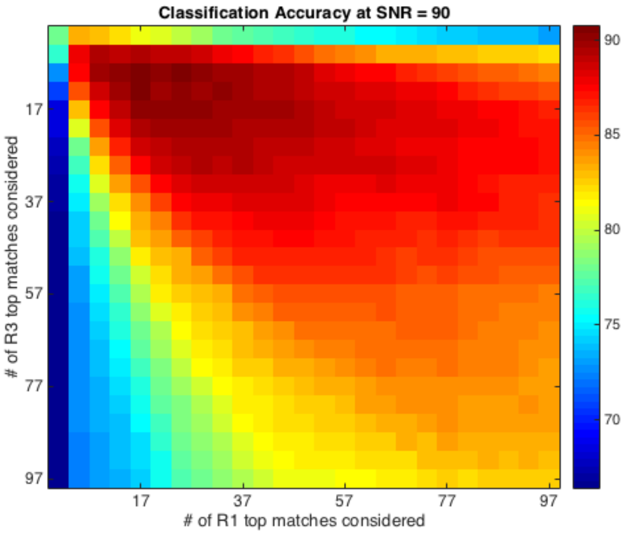

Supplementary Figure 1. Classification accuracy of our dictionary-based method as a function of the number of top matches used to calculate the cell similarity index. The number of top matches considered is different for each of the 4 dictionaries, resulting in a 4-dimensional parameter space. This figure shows the classification accuracy using the top 17 R2 and top 17 R4 matches to calculate the cell similarity index, with an image SNR = 90. The x- and y-axes show the variation in classification accuracy as the number of top R3 and R1 matches used in the calculation of the cell similarity index is varied. The maximum classification accuracy is reached when using the top (17, 17, 9, 17) matches in the (R1, R2, R3, R4) dictionaries to calculate the cell similarity index. The colourbar shows the percentage of cells correctly classified.

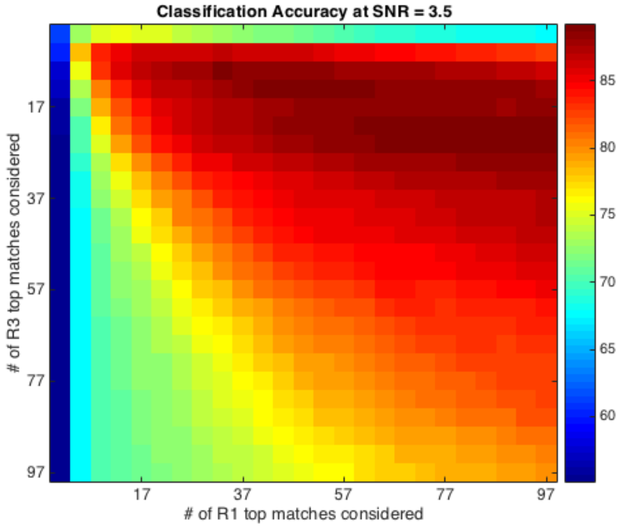

Supplementary Figure 2. Classification accuracy of our dictionary-based method as a function of the number of top matches used to calculate the cell similarity index. The number of top matches considered is different for each of the 4 dictionaries, resulting in a 4-dimensional parameter space. This figure shows the classification accuracy using the top 81 R2 and top 9 R4 matches to calculate the cell similarity index, with an image SNR = 3.5. The x- and y-axes show the variation in classification accuracy as the number of top R3 and R1 matches used in the calculation of the cell similarity index is varied. The maximum classification accuracy is reached when using the top (69, 81, 21, 9) matches in the (R1, R2, R3, R4) dictionaries to calculate the cell similarity index. The colourbar shows the percentage of cells correctly classified.

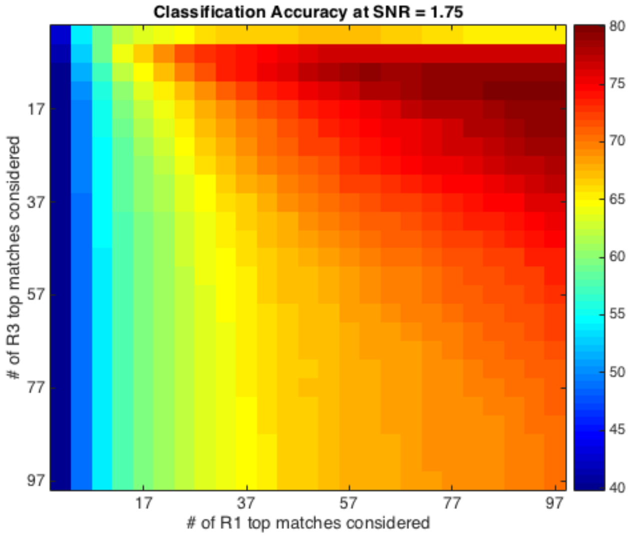

Supplementary Figure 3. Classification accuracy of our dictionary-based method as a function of the number of top matches used to calculate the cell similarity index. The number of top matches considered is different for each of the 4 dictionaries, resulting in a 4-dimensional parameter space. This figure shows the classification accuracy using the top 53 R2 and top 5 R4 matches to calculate the cell similarity index, with an image SNR = 1.75. The x- and y-axes show the variation in classification accuracy as the number of top R3 and R1 matches used in the calculation of the cell similarity index is varied. The maximum classification accuracy is reached when using the top (97, 53, 13, 5) matches in the (R1, R2, R3, R4) dictionaries to calculate the cell similarity index. The colourbar shows the percentage of cells correctly classified.

Supplementary Note 5

Classifying each trial cell involves calculating an image correlation between the trial cell and every cell in the dictionary. Though we only consider the top matches (most highly correlated dictionary cells), we have actually calculated thousands of correlation values. Supplementary Figure 4 shows the histogram of these correlation values for each of the correctly classified cells in Fig. 4 of the main text. Supplementary Figures 5-7 show the same data in a different format: the correlation value between the trial cell and dictionary cells is plotted as a function of the correlation value ranking (ie. the most dictionary cell with the highest correlation value is ranked 1 and so on).

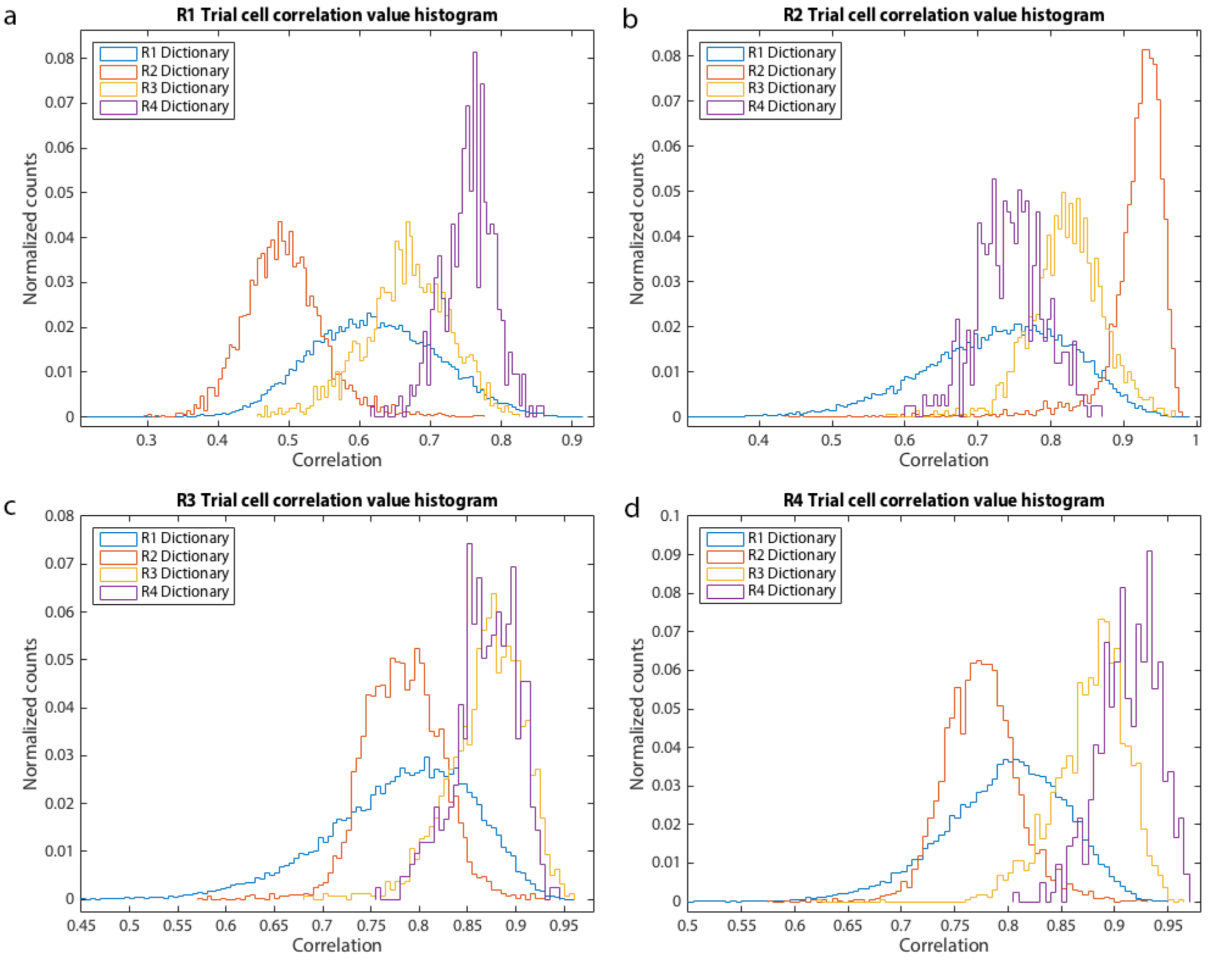

Supplementary Figure 4. Correlation histograms for the 4 correctly classified trials cells shown in Fig. 4. a-d: Correlation histograms for trial cells from gates R1-R4, respectively. Histograms are normalized so that they integrate to unity. The dictionary cells that determine classification are located on the high end of the correlation histogram (extreme right on the x-axis). Note that, in general, the mean value of the distributions is a poor indicator of cell classification. For example, the mean value of the R1 dictionary curve is lowest for the R1 trial cell. This is because there is a lot more morphological variability in R1 cells compared to the rest of the dataset. Despite this counterintuitive observation, the R1 trial cell is still correctly classified because of the long tail at the high correlation value side of the distribution. None of the other dictionaries contain a cell with a correlation value higher than 0.86 (the most highly correlated R4 cell), whereas the top 53 most correlated cells in the R1 dictionary have a correlation value higher than 0.86. The R2 dictionary histogram has a large right-shift towards higher correlations for the R2 trial cell, as expected due to the circular symmetry of the cells within this gate. Similar, though more subtle shifts also occur for R3 and R4 dictionary histograms for the R3 and R4 trial cells, respectively.

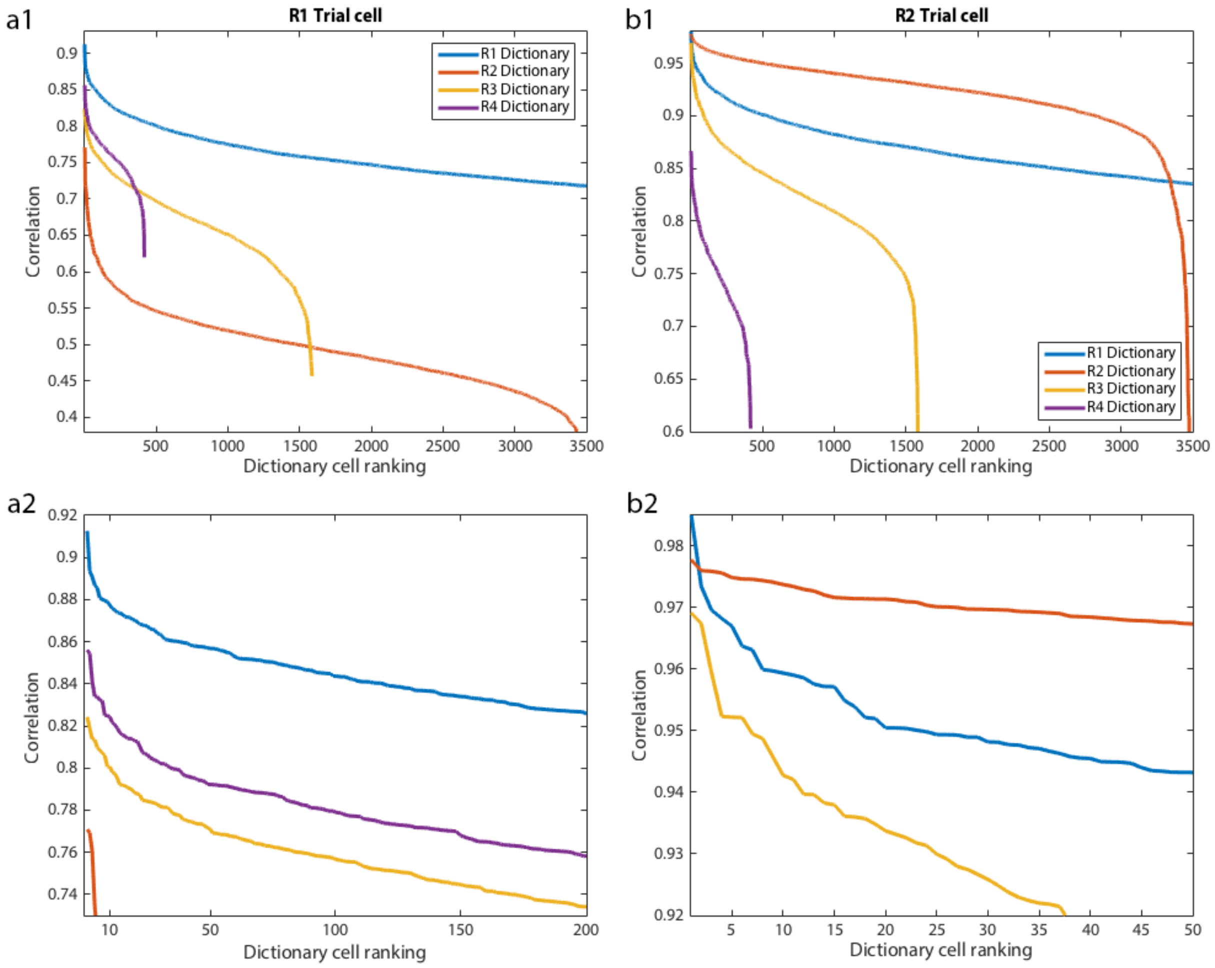

Supplementary Figure 5. Correlation value vs. dictionary cell ranking for R1 (a1 & a2) and R2 (b1 & b2) trial cells. The x-axis of each plot indicates ranking number of a given dictionary cell, with the most highly correlated cell being ranked 1. The y-axes indicate the correlation value of the dictionary cell of a given ranking with the trial cell. a1 & b1: Only the correlation values from the top 3500 matches from each dictionary are shown (curves for dictionaries with fewer than 3500 cells (R2-R4) have fewer than 3500 entries). Note the sharp increase in correlation values near the top ranked dictionary cells. The sharp decrease for low correlation dictionary cells is mostly due to small number of poorly segmented cells and debris. a2 & b2: Magnified versions of the a1 & b1 for the top 200 R1 (a2) and top 50 R2 (b2) dictionary cells. In a2, one can see that the top 53 R1 dictionary cells have a higher correlation value with the R1 trial cell than any cell in all other dictionaries. Thus, this trial cell is easily classified as R1. In b2 (for a R2 trial cell), the top ranked dictionary cell is from the R1 dictionary. However, the next 9 most highly correlated dictionary cells are all found in the R2 dictionary. Thus, the cell is correctly classified as R2, even though the most similar cell overall was found in the R1 dictionary. This example demonstrates why averaging over the top 10 correlation values is key. Using only the top ranked dictionary cell makes the approach more prone to accidental matching due to cross talk between dictionaries.

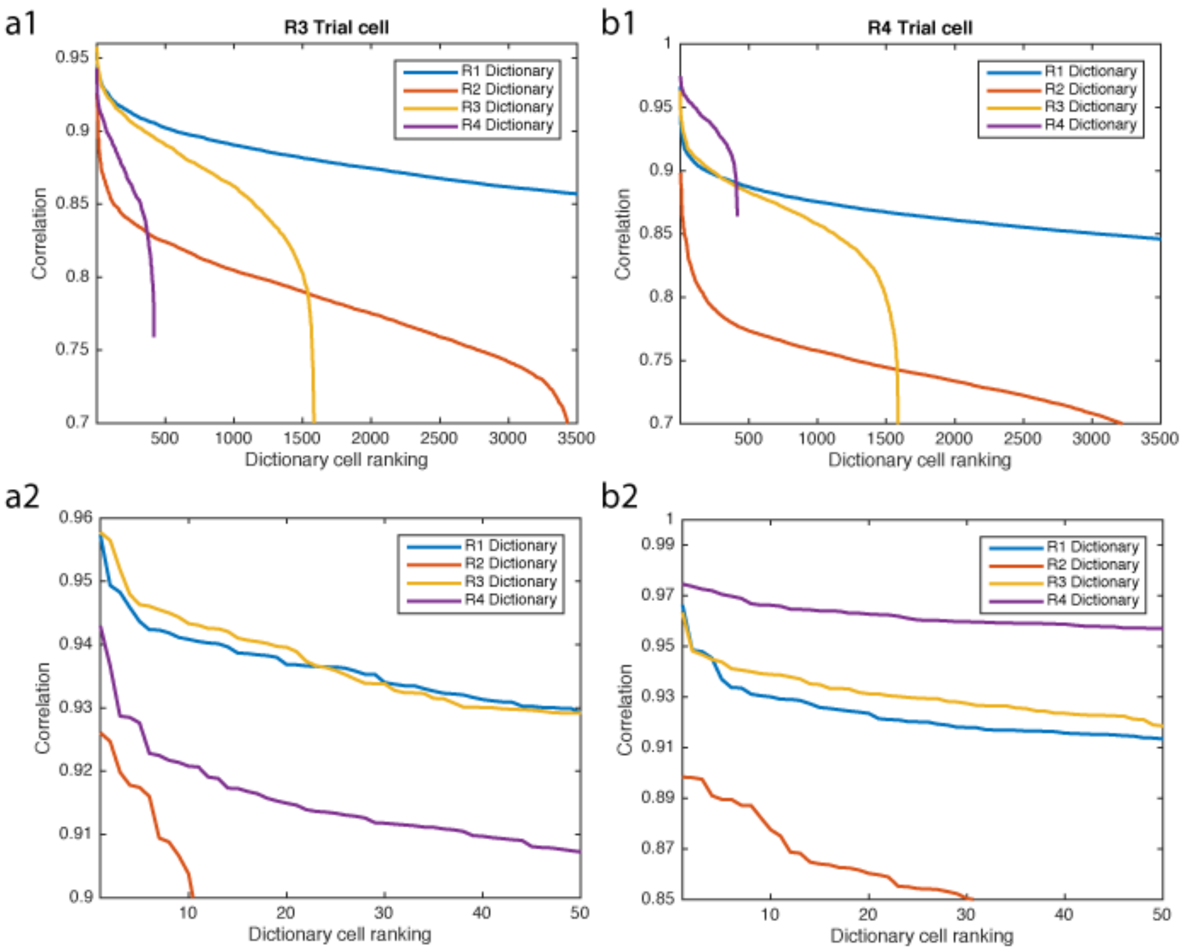

Supplementary Figure 6. Same as Supplementary Figure 5, except with R3 and R4 trial cells. These plots show correlation value vs. dictionary cell ranking for R3 (a1 & a2) and R4 (b1 & b2) trial cells.

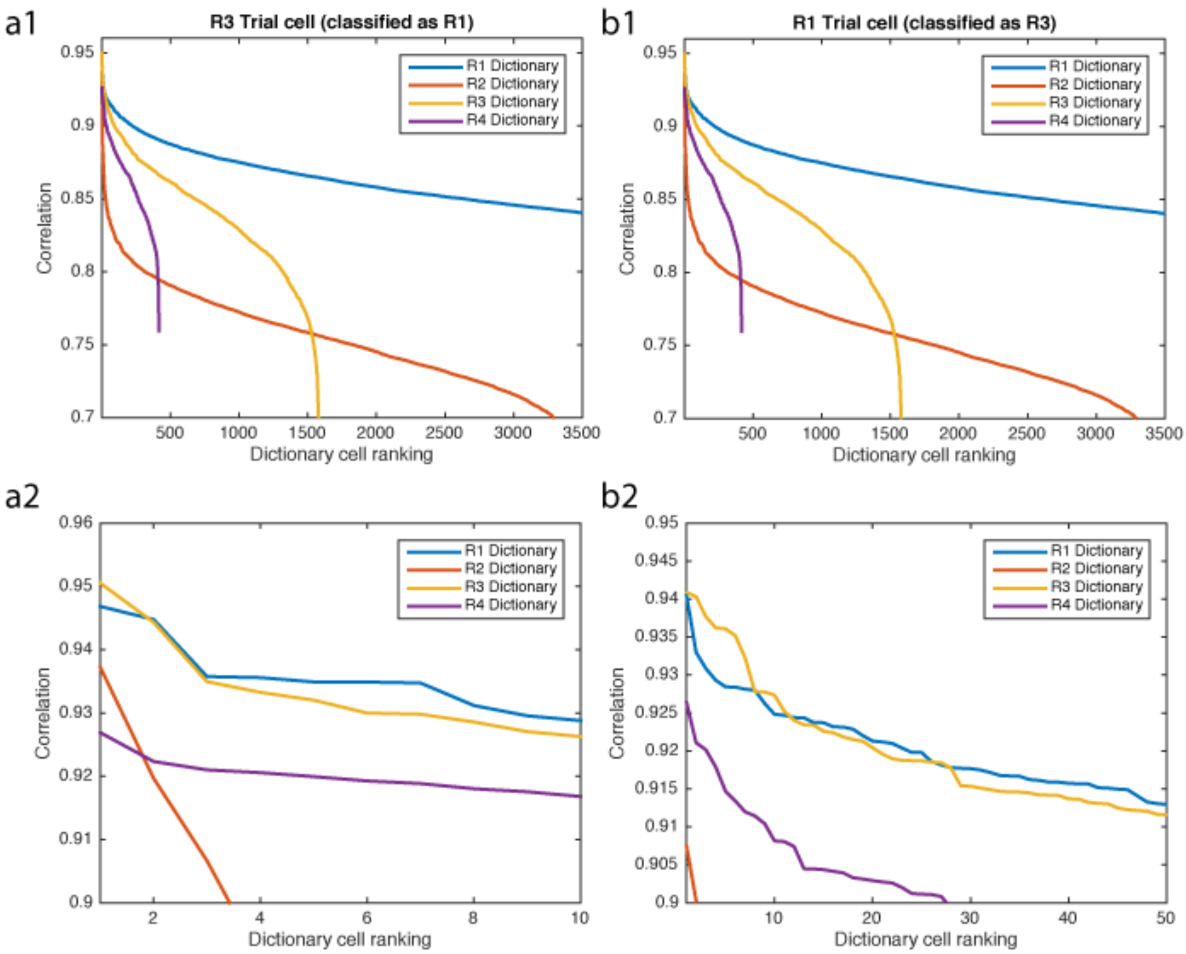

Supplementary Figure 7. Same as Supplementary Figures 5 and 6, but for an incorrectly classified R3 cell (classified as R1, a1 & a2) and an incorrectly classified R1 cell (classified as R3, b1 & b2).
